# Mouse PrimPol Outperforms Its Human Counterpart as a Robust DNA Primase

**DOI:** 10.3390/ijms26146947

**Published:** 2025-07-19

**Authors:** Gustavo Carvalho, Susana Guerra, María I. Martínez-Jiménez, Luis Blanco

**Affiliations:** Centro de Biología Molecular “Severo Ochoa” (CSIC-UAM), c/Nicolás Cabrera 1, 28049 Madrid, Spain; gustavo.carvalho@umu.se (G.C.); susanaguerrag@gmail.com (S.G.); maria_m@cbm.csic.es (M.I.M.-J.)

**Keywords:** PrimPol, mouse PrimPol, DNA primase, DNA replication

## Abstract

The human PrimPol counteracts DNA replication stress by repriming DNA synthesis when fork progression is hindered by UV light or hydroxyurea treatment, or by encountering complex DNA structures, such as G-quadruplexes, R-loops, or interstrand crosslinks. The *Mus musculus* PrimPol (*Mm*PrimPol) shares a high degree of amino acid similarity with its human ortholog; however, as shown here, *Mm*PrimPol exhibits a more powerful primase activity compared to the human enzyme. Such a robust primase activity relies on an enhanced ability to bind the 5′ site nucleotide, and consequently to form initial dimers and further mature primers. Additionally, a shorter linker between the AEP core and the Zn finger domain (ZnFD) in the murine homolog likely promotes a constitutive closing of these domains into a primase-ready configuration. Consequently, a reinforced close configuration of the ZnFD would explain why *Mm*PrimPol has a more robust primase, but a very limited DNA polymerization on an existing primer.

## 1. Introduction

Primases are enzymes responsible for initiating DNA replication, by making primers for the replicative DNA polymerases [1,2,3,4]. Humans possess two members of the archaeo-eukaryotic primases (AEP) family: the conventional dimeric primase (PriL–PriS), which is part of the Polα/primase complex and synthetizes RNA primers to initiate Okazaki fragments during lagging strand replication [5,6], and the monomeric PrimPol (*Hs*PrimPol), which has the ability to synthetize the DNA primers [7,8] that are essential for rescuing stalled replication forks during leading strand synthesis [9]. Both human primases share a high similarity in the catalytic domain. *Hs*PrimPol is characterized by three signature motifs: a highly conserved DxE motif (motif A, Asp^114^ and Glu^116^), which together with Asp^280^ residue from motif C, forms the divalent metal binding site [8,10,11], and motif B (formed by Lys^165,^ Ser^167^ and His^169^) along with Arg^291^ and Lys^297^ bind the incoming 3′ nucleotide mainly through its phosphates [11,12,13]. One specific difference between these two types of the AEP primases is that a single residue in PrimPols (Tyr^100^ in *Hs*PrimPol) contributes to nucleotide selectivity by acting as a steric gate that favors dNTP incorporation [14]. Additionally, the C-terminal domain of *Hs*PrimPol contains a highly conserved motif (Cys–X_5_–His–X_19_–Cys–X_4_–Cys), predicted to form a functional Zn-finger, which is essential for the *Hs*PrimPol primase activity [9,11,15,16], followed by two motifs that interact with the human single-stranded binding protein RPA [11,17].

The C-terminal domain containing the Zinc-finger (ZnFD) in eukaryotic PrimPols is highly similar to that found in the AEP primases from the nucleo-cytoplasmic large DNA virus family [7], such as herpes virus primase (UL52), which also requires its C-terminal ZnFD for primase activity [18,19]. A double point mutation in two of the Zn^2+^ ligands (C419G, H426Y) and a deletion mutant lacking the C-terminal domain (ΔZnFD, missing amino acids 410–560) were instrumental to demonstrate that this domain is involved in DNA priming, and that PrimPol’s primase function is required in vivo to re-start stalled replication forks [9]. On the other hand, the ZnFD is not necessary for conventional DNA polymerase activity, or for translesion synthesis (TLS) during primer extension [9,15].

**Figure 1:**
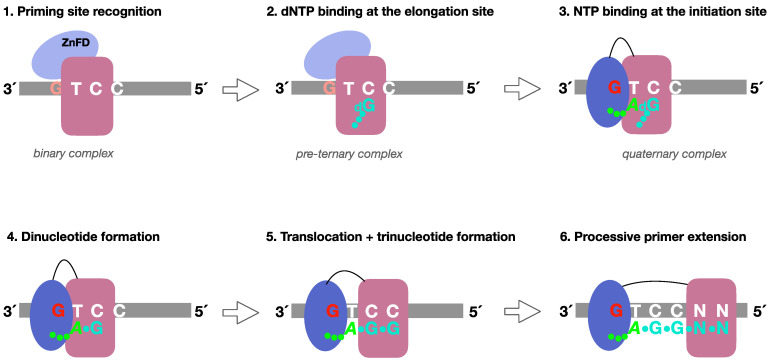
Mechanism of DNA priming by the human PrimPol. The schematics delineate the sequential steps during primer synthesis by *Hs*PrimPol. (1) Recognition of a preferred priming site: Binding of PrimPol to ssDNA does not rely on the G (in orange) of the preferred 3′..**G**TC..5′ initiation site, and does not require the Zn-finger domain (ZnFD, light blue). (2) Deoxynucleotide binding at the 3′-elongation site: The 3′-deoxynucleotide (dGTP, cyan) binds to the elongation site without the influence of the ZnFD, but requires manganese ions and template base-complementarity with the **C** in the 3′..GT**C**..5′ template sequence to stabilize such a pre-ternary complex. (3) Nucleotide binding at the 5′-initiation site: The 5′-ribonucleotide (*ATP*, green) binds opposite the T in the 3′..G**T**C..5′ template sequence in an optimal configuration facilitated by the G (now in red), and the ZnFD (now in dark blue) that binds both the G and the triphosphate moiety of the initiating 5′-nucleotide. (4) Dinucleotide formation: Such a closed conformation activates catalysis, promoting formation of a *_3p_A*-G dinucleotide. (5) Translocation and trinucleotide formation: The ZnFD maintain the interaction with the G at the 3′..**G**TC..5′ template sequence and with the 5′-end triphosphate, facilitating dimer translocation and next nucleotide addition. (6) Processive elongation: The primer is elongated processively at least up to 10 nucleotides, likely favored by a persisting interaction of the ZnFD with the 5′-triphosphate at the end of the primer, and by its flexible tethering to the catalytic core of PrimPol. [16].

The initial steps of primer synthesis by *Hs*PrimPol and the role of the ZnFD at each individual step were delineated as follows [16] (Figure 1, see legend for further details):

(1) Formation of a PrimPol/ssDNA binary complex.
(2) Further interaction with the incoming 3′-deoxynucleotide (pre-ternary complex).
(3) The ZnFD is mobilized to facilitate binding and selection of the 5′-nucleoside triphosphate (preferentially a ribonucleotide), that will become the first nucleotide of the newly synthesized primer.
(4) Once the 5′-nucleotide (acting as a primer) and the 3′-nucleotide are selected and a quaternary complex is formed, catalysis of the initial dimer occurs.
(5) A maintained interaction of the ZnFD with the 5′-terminal triphosphate is essential for the subsequent translocation and insertion event to form a trimer.
(6) Further elongation of the primer occurs processively, i.e., with no dissociation, thanks to the sustained interaction of the ZnFD with the initiating nucleotide. When the primer reaches an optimal length, PrimPol dissociates and the primer is extended by the replicative DNA polymerase.

The in vivo role of the human PrimPol has been mainly determined using human cell lines [20,21,22,23] but also taking advantage of chicken DT40 cells [24] and even a mouse model of PrimPol deficiency. Our laboratory established a C57BL/6 mice colony with a knock-out for the CCDC111 gene, encoding PrimPol. This CCDC111–/– colony was very relevant to study the physiological role of PrimPol, and to generate mouse embryonic fibroblasts (MEFs) for in vivo assays that revealed its nuclear and mitochondrial functions [8,9,14,25]. Strikingly, mouse cells lacking the endogenous murine PrimPol were poorly rescued by ectopic expression of the human PrimPol ortholog [14] suggesting that the two PrimPols may have important differences in their enzymatic activity. Therefore, and because only the activity of the human PrimPol has been described to date, we aimed to study the enzymatic activities of the mouse PrimPol. As shown here, *Mm*PrimPol and *Hs*PrimPol share similar characteristics, but unexpectedly large differences in specific activity. *Mm*PrimPol exhibits a robust primase activity, also dependent on the ZnFD, and a poor DNA polymerase activity, which further supports PrimPol’s physiological relevance as a primase, and suggests that the constitutive configuration of *Mm*PrimPol is ready to prime. Cross-species comparisons provide invaluable insights into enzyme evolution, mechanistic diversity, and physiological adaptation. This study exemplifies how such analyses can uncover both conserved and specialized functions, advancing our understanding of DNA replication across organisms.

## 2. Results

### 2.1. The Mouse and Human PrimPol Share High Amino Acid Sequence Similarity

**Figure 2:**
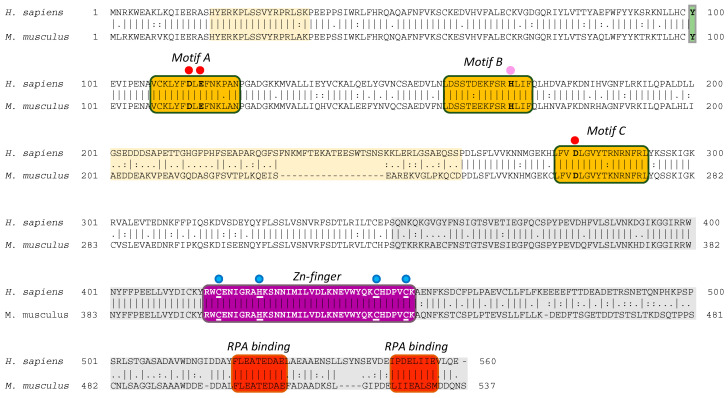
The human and mouse PrimPol are highly similar, as evidenced by amino acid sequence comparisons. The degree of amino acids similarity is indicated as (|) for identical, (:) for conservative, and (.) for semi-conservative. Red dots indicate the metal ligands Asp^114^ and Glu^116^ in motif A, and Asp^280^ (human) or Asp^262^ (mouse) in motif C. The pink dot indicates the nucleotide ligand His^169^ (motif B). The C′2-OH sugar selector Tyr^100^ is shown in a green box. The region forming a Zn-finger (purple box) contains four conserved cysteines and one histidine (blue dots). Two RPA binding motifs (red boxes) are localized at the C-terminus. The crystal structure of *Hs*PrimPol^(1–354aa)^ [26] does not contain the C-terminal region indicated with grey boxes, and presents two unresolved segments, indicated with light yellow boxes.

*Mm*PrimPol is codified by the *CCDC111* gene, which is located on chromosome 8 (8 B1.1) and spans over 40 kb (NCBI Gene ID: 408022). It is transcribed into a 3835 bp mRNA (NCBI Reference sequence: NM_001001184.2) which expresses a 537- amino acid polypeptide (NCBI Ref. seq.: NP_001001184.1) with a predicted molecular weight of 61.34 kDa. Amino acid sequence alignments have revealed an 80% similarity between the human and mouse PrimPols (Figure 2). Like *Hs*PrimPol, *Mm*PrimPol contains the conserved catalytic motifs A, B, and C characteristic of the AEP primases, along with a highly conserved Zinc-finger and two C-terminal RPA binding motifs. As expected, the catalytic residues of Asp^114^ and Glu^116^ (DxE) in motif A of *Hs*PrimPol, which serve as metal ligands, are invariantly conserved in *Mm*PrimPol; in addition, the third catalytic residue, Asp^262^ in *Mm*PrimPol (corresponding to Asp^280^ in *Hs*PrimPol), located in motif C is also conserved (indicated with red dots in Figure 2). The His^169^ residue in motif B, critical for binding the incoming 3′-nucleotides [11,12,26], is also conserved in both PrimPols (Figure 2). The carboxy terminal region of both PrimPols contains a Zn-finger, formed by three cysteines and one histidine (Cys–His–Cys–Cys, indicated by blue dots in Figure 2) with the potential to coordinate a Zn^2+^ atom, which is 100% identical in both PrimPols. Downstream of the ZnFD, two RPA binding sites (indicated by red boxes in Figure 2) are also 100% identical between both *Hs*PrimPol and *Mm*PrimPol.

A multiple sequence alignment of PrimPols from diverse organisms [8] identified a variable region situated between the catalytic motifs B and C. This region is moderately conserved and varies in length, being approximately 50 residues longer in mammals compared to insects or plants. Further alignments of mammalian PrimPols performed in this study indicate that only the Muridae family has lost an 18 aa sequence within this variable region, corresponding to amino acids 229 to 246 in *Hs*PrimPol (Figure 2 and Figure 9). Interestingly, this variable region (residues 201 to 260, indicated by light yellow in Figure 2) was disordered in the crystal structure of *Hs*PrimPol [26].

### 2.2. The Mouse PrimPol Is a True DNA Primase

**Figure 3:**
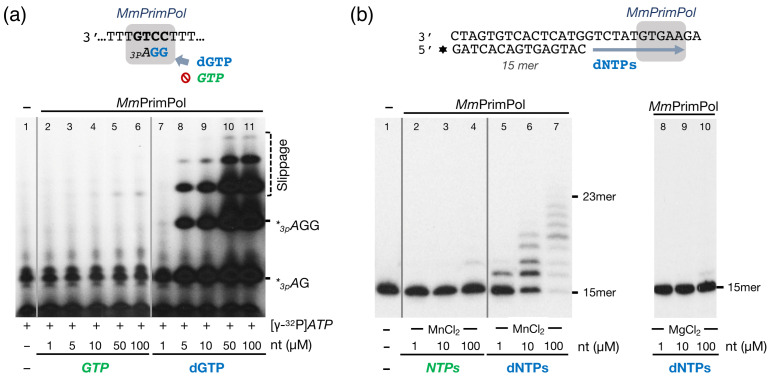
The mouse PrimPol is a true DNA primase. (**a**) Primase activity of *Mm*PrimPol (400 nM) was assessed in the presence of 1 µM 3′ T_10_-GTCC-T_15_ 5′ as template, 1 mM MnCl_2_, 16 nM of the 5′-initiating [γ-^32^P]*ATP*, and increasing concentrations of either *GTP* or dGTP, as shown in the figure; in addition to the expected canonical products (*_3p_A*dG and *_3p_A*dGdG), other longer but non canonical products observed (slippage) are due to reiterative insertion of dGs by backwards slippage of the primer over the CC template sequence. (**b**) DNA polymerization by *Mm*PrimPol (200 nM) was assayed using a pre-synthesized primer (15mer) hybridized to a template (2.5 nM) containing a 13-nt overhang, 1 mM MnCl_2_ or 5 mM MgCl_2_ was used as metal cofactor, and a mixture of the four *NTPs* or dNTPs at the indicated concentrations.

*Hs*PrimPol preferentially uses deoxynucleotides (dNTPs) over ribonucleotides as substrates at the elongation site for both primase and polymerase activities, unlike conventional RNA primases [8]. This preference for dNTPs is primarily attributed to *Hs*PrimPol Tyr^100^, which acts as the sugar selector for incoming 3′-nucleotides; in fact, a mutation of Tyr^100^ to histidine inverts this selectivity, now allowing for the incorporation of ribonucleotides by *Hs*PrimPol [14]. Given that Tyr^100^ is conserved in both the human and the mouse PrimPol, we presumed that *Mm*PrimPol would also be biased to use dNTPs as 3′-incoming nucleotides. DNA primase activity was firstly assessed by using as a template the 29-mer single-stranded pyrimidine-rich oligonucleotide 3′ T_10_-GTCC-T_15_ 5′ (hereafter referred to as 3′ GTCC 5′). This 3′GTC sequence was previously identified as a preferred initiation site for *Hs*PrimPol using in vitro assays [8], and as a favored template context for various viral, prokaryotic, and eukaryotic RNA primases [27], thus predicting its suitability for measuring the *Mm*PrimPol primer synthesis. In addition to the 3′ GTCC 5′ template, a low concentration (16 nM) of [γ-^32^P]*ATP* was provided as the initiating 5′-nucleotide, to be positioned opposite the template’s T (G**T**CC), and increasing concentrations of *GTP* or dGTP as the only 3′-nucleotide, to be inserted opposite the two consecutive Cs in the template (GT**C****C**), thus forming di- and trinucleotide products, as outlined in the scheme in Figure 3a. Manganese ions (1 mM) were supplied as the preferred metal co-factor for PrimPol activity. As expected, *Mm*PrimPol was unable to initiate a de novo synthesis of RNA primers when *ATP* and *GTP* were supplied as substrates, even with *GTP* concentrations up to 100 µM (Figure 3a, lanes 2–6); however, *Mm*PrimPol exhibited robust DNA primase activity when dGTP was provided as the 3′-nt, synthesizing *_3p_A*G dimers when dGTP was supplied at a concentration as low as 5 µM (Figure 3a, lanes 8–11). Increasing dGTP concentrations led to the production of canonical trimers (*_3p_A*GG), and to longer non-canonical products (such as *_3p_A*GGG or *_3p_A*GGGG), obtained by backwards slippage of the last incorporated G to be realigned with the first C in the template, yielding to the iterative insertion of additional Gs opposite the second consecutive C of the template, as previously described for the human PrimPol [8].

The sugar selectivity of *Mm*PrimPol was further explored during a conventional polymerization assay. For this, a pre-synthesized DNA primer was hybridized to a DNA template with a 13-nt overhang, and polymerization was tested in a reaction mixture containing the four *NTPs* or dNTPs. As shown in Figure 3b (lanes 2–4), no primer extension (polymerization) by *Mm*PrimPol was observed with *NTPs* as 3′-substrates, consistent with the primase assay. Conversely, *Mm*PrimPol extended the pre-synthesized DNA primer in the presence of dNTPs and MnCl_2_, with products increasing in yield and length in response to the concentration of dNTPs provided (Figure 3b, lanes 5–7). This confirms *Mm*PrimPol’s preference for dNTPs at the 3′-elongation site, but the limited elongation pattern reflects a modest DNA polymerase activity. As other groups have reported that *Hs*PrimPol can utilize magnesium as catalytic metal [11,28,29,30], we tested if *Mm*PrimPol could be using magnesium to activate DNA polymerization. *Mm*PrimPol showed a null polymerization capacity (Figure 3b, lanes 8–10), indicating that magnesium is not a suitable metal co-factor for *Mm*PrimPol (at least, under our experimental conditions).

Collectively, these preliminary experiments suggested an altered balance of Primase/Polymerase activities in *Mm*PrimPol compared to *Hs*PrimPol [8].

### 2.3. The Mouse PrimPol Has a Strong Preference to Initiate and Complete Primer Synthesis, but Not to Extend Pre-Existing Primer Chains

**Figure 4:**
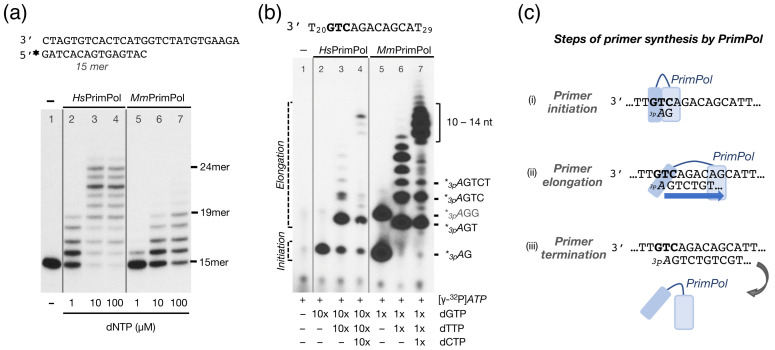
Mouse and human PrimPols exhibit an inverted balance of DNA polymerization and DNA primase activities. (**a**) DNA polymerase activity of *Hs*PrimPol (200 nM) or *Mm*PrimPol (200 nM) was assayed in the presence of 1 mM MnCl_2_, a pre-synthesized [γ-^32^P]-labeled primer (15mer) hybridized to a template containing a 13-nt overhang, and increasing concentrations of dNTPs. The length of some elongation products is indicated. (**b**) DNA primase activity was assessed in the presence of the 3′ T_20_**GTC**AGACAGCAT_29_ 5′ template, [γ-^32^P]*ATP* (16 nM), and sequential addition of dGTP, dTTP and dCTP. Whereas, *Hs*PrimPol (400 nM) initiated primer synthesis (formation of _3p_*A*G) by providing a moderate amount of dGTP (10 µM, indicated as 10×), *Mm*PrimPol (400 nM) was much more efficient, forming a much higher amount of initiating dimers even by providing a 10-fold lower concentration of dGTP (1 µM, indicated as 1×). In the presence of the remaining dNTPs, both the human and the mouse PrimPols elongated their newly-initiated dimers, accumulating products of 10–14-nt long (lanes 4 and 7). (**c**) Scheme of PrimPol primer synthesis: (i) synthesis of a dimer using preferably two purines, (ii) elongation of the dimer generating longer and non-abortive products, and (iii) termination of primer synthesis and dissociation of PrimPol from the primer–template structure. The scheme emphasizes the contribution of the ZnFD (dark blue square) to both initiation and elongation steps of primer synthesis by the AEP domain (light blue square).

After demonstrating that *Mm*PrimPol has a strong capacity to initiate DNA primers, but a very limited capacity as a DNA polymerase, the balance of both activities was comparatively tested between *Hs*PrimPol and *Mm*PrimPol. As shown in Figure 4a, *Hs*PrimPol was significantly more efficient (more than 10-fold) than *Mm*PrimPol in a conventional DNA polymerization assay (qualitatively estimated to be more than 10-fold; note the same elongation pattern observed with the two PrimPols at different (10-fold) dNTP concentration: lane 2 vs. lane 6, and lane 3 vs. lane 7). For a complete evaluation of the DNA primase activity, it is important to consider that, during de novo primer synthesis, a primase has to encompass both initiation (dinucleotide formation) and its further elongation (extending the dimer to a 7–10 nucleotide primer). Given *Mm*PrimPol’s low DNA polymerase activity, we investigated whether this would impair the elongation step of primer synthesis, leading to the generation of only short, aborted primers. To address this, we designed a longer ssDNA template containing PrimPol’s favorite priming sequence, 3′ **GTC** 5′, followed by a heterogeneous 8 nt sequence (3′ T_20_**GTC**AGACAGCAT_29_ 5′ called 3′ GTC-step 5′). This template allowed us to explore the initiation and elongation process of primer synthesis by *Mm*PrimPol in a single assay. To delineate the sequential steps (initiation and elongation) when priming on the 3′ **GTC**-step 5′ template, we provided a low dose (16 nM) of the initiating 5′-nucleotide [γ-^32^P]*ATP* to radioactively label all nascent products, and complementary 3′-dNTPs were consecutively added to the reaction, to follow the sequential products of primer synthesis (*_3p_A*G, *_3p_A*GT, *_3p_A*GTC, and so on…). Of note, due to its robust DNA primase activity, *Mm*PrimPol was assayed with a 10-fold lower dNTP concentration than *Hs*PrimPol.

Upon addition of the first two nucleotides, *ATP* and dGTP, both *Hs*PrimPol and *Mm*PrimPol successfully initiated primer synthesis, generating *_3p_A*G dimers (Figure 4b, lanes 2 and 5). This indicates that both enzymes recognized their favorite priming site (GTC**)** on the new oligo sequence. Despite the 10-fold lower dGTP concentration used, the *Mm*PrimPol primase activity resulted in a much higher formation of the *_3p_A*G dimer, and even the *_3p_A*GG trimer, likely through repetitive insertion of a second dGMP unit by slippage (Figure 4b, lane 5). With the addition of the next nucleotide (dTTP), *Hs*PrimPol primarily produced the expected trimer (*_3p_A*GT), alongside some residual dimers and longer unscheduled products; whereas, *Mm*PrimPol efficiently converted all dimers into trimers or longer primers (Figure 4b, lanes 3 and 6), the latter arising from misalignment events [12]. Finally, upon addition of a fourth nucleotide (dCTP), both PrimPols elongated their newly synthesized dimers and trimers, generating canonical extension products up to 10 to 14-nt long (Figure 4b, lanes 4 and 7). Thus, in stark contrast to our previous DNA polymerase assay (Figure 4a), *Mm*PrimPol was perfectly capable of elongating its newly initiated primers, performing even better than *Hs*PrimPol, despite the 10-fold lower total dNTP concentration. These results demonstrate that *Mm*PrimPol effectively carries out “polymerization” when elongating its self-generated primers, but is very deficient to extend a pre-existing primer (unlike conventional DNA polymerases do). These results reinforce the notion that the important function of PrimPols is their DNA primase activity, and points to the Zn-finger of *Mm*PrimPol as the main responsible for its robust DNA primase activity.

### 2.4. The Zinc-Finger Domain of the Mouse PrimPol Brakes Its DNA Polymerase Activity but Is Essential for Its Strong DNA Primase Activity

**Figure 5:**
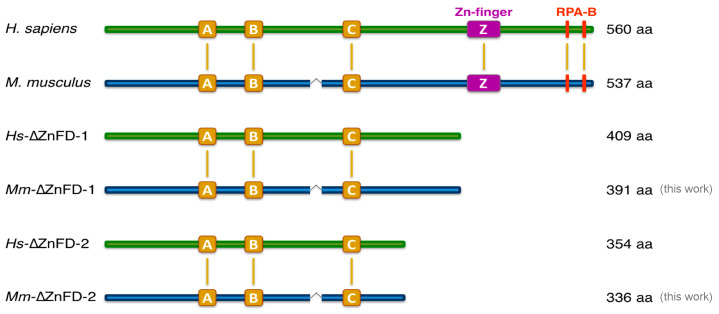
Generation of the *Mm*PrimPol Zn-finger domain deletion mutants. Modular organization of PrimPols from *Homo sapiens* and *Mus musculus* showing the corresponding wild-type proteins and the mutants lacking the Zn-finger domain (∆ZnFD). *Hs*PrimPol mutants, *Hs*-∆ZnFD-1^(1–409)^ and *Hs*-∆ZnFD-2^(1–354)^, were used as references for the design of the two murine mutants *Mm*-∆ZnFD-1^(1–391)^ and *Mm*-∆ZnFD-2^(1–336)^.

The AEP core of *Hs*PrimPol has been crystallized, but is lacking the C-terminal part which includes the Zinc-finger domain (ZnFD) and the RPA binding motifs [26]. Consequently, our understanding of the ZnFD primarily comes from in vitro biochemistry assays and in vivo studies using deletion or point mutants. Previously, we demonstrated that the ZnFD of *Hs*PrimPol is essential for primase activity by eliminating this entire domain, along with the downstream C-terminal region (*Hs*∆ZnFD1^(1–409)^) of *Hs*PrimPol [9,16] (Figure 5). Conversely, the *Hs*∆ZnFD-1 displayed a significantly enhanced DNA polymerase activity, indicating that the ZnFD inhibits extension of a pre-existing primer. Given the altered balance between the primase and polymerase activities in *Mm*PrimPol, we questioned whether the elimination of the Zn-finger would also be crucial for the primase activity, and could similarly enhance its DNA polymerase activity. To investigate this, we generated two *Mm*PrimPol mutants (Figure 5) by deleting the ZnFD The first mutant, *Mm*∆ZnFD-1^(1–391)^, resembling our previously studied *Hs*∆ZnFD-1^(1–409)^, and obtained by introducing a stop codon immediately after the conserved sequence FFPEELL, localized 7 amino acids upstream the ZnFD in both the human and the mouse PrimPols (Figure 5). The second deletion mutant of *Mm*PrimPol, *Mm*∆ZnFD-2^(1–336)^, which preserves the semi-conserved sequence PSQTKRK (amino acids 330–336 of *Mm*PrimPol; the amino acids that differ from *Hs*PrimPol are underlined), and corresponds to the crystalized form of *Hs*PrimPol *Hs*∆ZnFD-2^(1–354)^ [15,26] (Figure 5).

**Figure 6:**
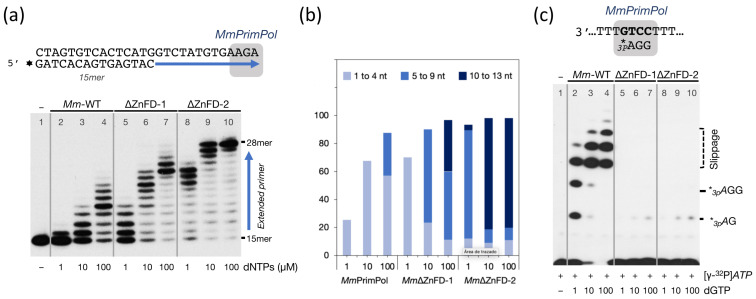
*Mm*PrimPol mutants lacking the ZnFD become DNA polymerase-proficient but DNA primase-dead. (**a**) DNA polymerase activity of the wild-type and ∆ZnFD *Mm*PrimPol were evaluated using a pre-synthesized [γ-^32^P]-labeled primer (15mer) hybridized to a template containing a 13-nt 3′-overhang, and increasing doses of dNTPs as indicated. (**b**) Quantification of the extent of primer extension in the DNA polymerase assay shown in (**a**). The ZnFD deletion mutants *Mm*-∆ZnFD-1 and *Mm*-∆ZnFD-2 increased DNA polymerization by a factor of approximately 10- and 100-fold, respectively. (**c**) DNA primase activity of the wild-type *Mm*PrimPol and the mutants *Mm*-∆ZnFD-1 and *Mm*-∆ZnFD-2, using the preferred 3′ T_10_-GTCC-T_15_ 5′ template, in the presence of 16 nM [γ-^32^P]*ATP* and increasing concentrations of dGTP, as indicated. Incipient primers generated in these reactions are indicated on the right border of the gel, and correspond to both canonical (*_3p_A*G and *_3p_A*GG) and slippage-mediated products. The robust DNA primase activity of *Mm*PrimPol is lost when deleting the ZnFD.

The impact of ZnFD elimination was firstly examined by re-evaluating the limited DNA polymerase activity of *Mm*PrimPol when extending a pre-synthesized primer (Figure 6a). Notably, both ZnFD-deletion mutants of *Mm*PrimPol significantly stimulated its polymerization activity, generating longer products compared to the WT *Mm*PrimPol (Figure 6a,b), and mimicking the increase in the polymerase activity reported for *Hs*PrimPol as a consequence of deleting its ZnFD [9]. The deletion mutants generated longer products compared to the WT *Mm*PrimPol (Figure 6a,b); moreover, a direct correlation was observed between the extent of the ZnFD deletion and the gain in DNA polymerase activity. Collectively, these findings indicated that the poor DNA polymerase activity of *Mm*PrimPol is not attributable to a catalytic deficiency, but rather to an inhibitory effect exerted by its ZnFD. Such an inhibition is likely the consequence of a more closed configuration of both the ZnFD and the AEP, that impedes binding to a preexisting primer, but is more prone to initiate a *de novo* primer.

In agreement with this hypothesis, both ZnFD-depleted mutants *Mm*∆ZnFD-1 and *Mm*∆ZnFD-2 failed to initiate de novo primer synthesis, in stark contrast with the strong primase activity showed by the wild-type *Mm*PrimPol (Figure 6c). This indicates that the ZnFD of *Mm*PrimPol is essential for its strong DNA primase activity, in good agreement with the data reported for *Hs*PrimPol [9,15,16].

### 2.5. The Mouse and Human PrimPols Form Similar Binary and Pre-Ternary Complexes as Primase Intermediates

To elucidate the basis for the robust primase activity observed in *Mm*PrimPol, a detailed investigation of the individual steps leading to primer synthesis is essential. As previously proposed by Sheaff and Kuchta (1993) [31], primer synthesis proceeds through the following series of defined stages: formation of an enzyme/ssDNA binary complex; followed by the binding of the first nucleotide at the 3′-elongation site of the primase (pre-ternary complex); followed then by the binding of a second nucleotide at the 5′-initiation site (quaternary complex), leading to subsequent catalysis, which renders dimer formation. These fundamental steps have been well-defined for *Hs*PrimPol [16].

**Figure 7:**
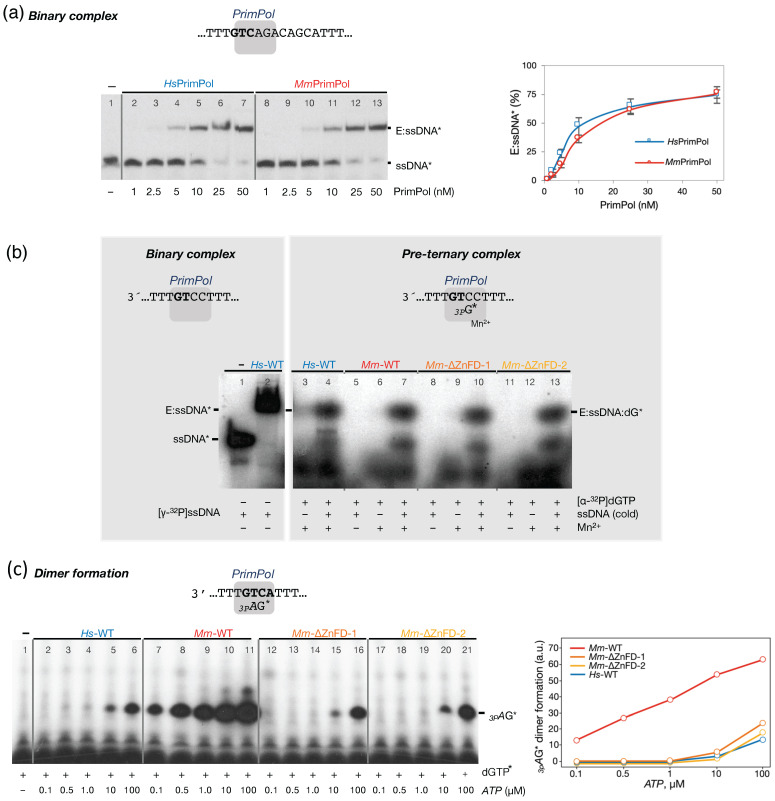
The robust *Mm*PrimPol primase activity does not rely on a better formation of binary and pre-ternary complexes, but in a higher affinity for the initiating 5′-nucleotide. (**a**) Electrophoresis mobility shift assay (EMSA) of the human and mouse PrimPol with the [γ-^32^P]-labeled oligonucleotide 3′ T_20_**GTC**AGACAGCAT_29_ 5′ showed that both primases can bind ssDNA with the same affinity. Quantification of the binary complex is shown at the right. (**b**) Binary complex (E/ssDNA*) of *Hs*PrimPol and [γ-^32^P]-labeled oligonucleotide 3′ T_20_-GTCC-T_36_ 5′ (lane 2) was included as a mobility control in the EMSA assay. As shown at the right part of the same autoradiogram, similar amounts of the pre-ternary complex (PrimPol/ssDNA/dGTP*), running as a single retarded band and with a similar mobility than that of the binary complex (lane 2), were formed when providing unlabeled ssDNA 3′ T_20_-GTCC-T_36_ 5′ (1 µM), [α-^32^P]dGTP as the 3′-nucleotide, 1 mM MnCl_2_, and either the human (lane 4), the mouse PrimPol (lane 7), or the ZnFD-deleted versions of the mouse PrimPol (lanes 10 and 13) (0.5 µM), indicating that the efficiency to form a pre-ternary complex by *Mm*PrimPol does not explain its robust primase activity, and it does not require the presence of a Zn-finger domain. Note that the mobility of the pre-ternary complex formed *Mm*PrimPol variants in this EMSA analysis is not affected by deleting its ZnFD (compare lane 7 with lanes 10 and 13), as it was previously reported for the human PrimPol [16]. In the absence of Mn^2+^ ions, no pre-ternary complex was formed (lanes 5, 8, and 11). (**c**) The _3p_*A*G dimer formation by the human and mouse PrimPol (400 nM) was assessed providing oligonucleotide 3′ T_10_-GTCA-T_15_ 5′ (1 µM) as a template, 1 mM MnCl_2_, low concentration (16 nM) of the 3′-elongating nucleotide [α-^32^P]dGTP, and increasing concentrations of the 5′-initiating nucleotide *ATP*. The wild-type *Mm*PrimPol presented a very efficient use of the 5′-initiating ribonucleotide (*ATP*) compared to the ∆ZnFD mutants as well as with the WT *Hs*PrimPol. Quantification of the *_3p_A*G dimers formed at different *ATP* concentrations is shown on the right. Note: a.u. stands for arbitrary units, derived from densitometric analysis of the autoradiographs.

Consequently, the individual steps required for primer initiation by *Mm*PrimPol were studied, in comparison with its human ortholog. Firstly, we assessed the capacity of *Mm*PrimPol to form a binary complex with an ssDNA template (3′ T_20_**GTC**AGACAGCAT_29_ 5′) using EMSA assays. Both the human and the mouse PrimPol formed a single E/ssDNA binary complex, at similar protein/ssDNA ratios (Figure 7a), therefore suggesting a similar affinity for ssDNA. Next, we investigated the binding of [α-^32^P]dGTP (hereafter referred as dGTP*) at the 3′site of both PrimPols in the presence of ssDNA (pre-ternary complex). Upon providing both the ssDNA template 3′ **GTC**C 5′ and dGTP*, the labeled deoxynucleotide is expected to occupy the elongation site, opposite the dC at the 3′GT**C**5′ priming site. Paradoxically, the first nucleotide bound by PrimPol (and other primases) ultimately becomes the second nucleotide in the newly synthesized dimer. As shown by the EMSA assays, a PrimPol/ssDNA/dGTP* pre-ternary complex (Figure 7b) was formed with either the human or the mouse PrimPol with similar efficiency, revealing that the binding of the 3′-dNTP at the elongation site does not account for the robust priming activity of *Mm*PrimPol. The pre-ternary complex (*Mm*PrimPol/ssDNA/dGTP*) was formed only in the presence of Mn^2+^ ions (Figure 7b), underscoring the crucial role of the activating metal for 3′dNTP binding. An analysis of the two *Mm*∆ZnFD deletion mutants demonstrated their ability to form the pre-ternary complex (Figure 7b). These results allow us to conclude that the murine ZnFD is not required for the binding of the incoming 3′-dNTP, also in agreement with previous studies with *Hs*PrimPol [16].

### 2.6. The Zn-Finger Domain of the Mouse PrimPol Confers High Affinity for the Initiating 5′-Ribonucleotide

Having discarded a differential interaction of *Mm*PrimPol and *Hs*PrimPol with ssDNA and the 3′-site dNTP, the robust primase activity displayed by *Mm*PrimPol is still not understood. This led us to investigate whether this robustness relays on the use of the 5′-site nucleotide during primer synthesis initiation. To explore this alternative, we performed DNA primase assays with the template oligonucleotide 3′ T_10_G**TC**AT_15_ 5′ to limit the synthesis to a *_3p_A*G dimer. Reactions were set up with a low concentration (16 nM) of the 3′ site nucleotide [α-^32^P]dGTP and increasing doses of the initiating 5′ site nucleotide, *ATP*. Our findings revealed that the potent primase activity of *Mm*PrimPol is likely driven by an exceptionally high affinity for the 5′-nt. Remarkably, *Mm*PrimPol synthesized much higher amounts of dimer than *Hs*PrimPol, requiring approximately 500–1000-fold lower *ATP* concentrations to produce the same amounts of dimers (Figure 7c, compare lanes 6 and 7). This new experiment confirmed the critical role of the ZnFD of *Mm*PrimPol in dimer formation, as a 500- to 1000-fold higher concentration of *ATP* was required to produce similar dimers when the ZnFD of *Mm*PrimPol was deleted (compare lanes 7, 16, and 21), suggesting that the ZnFD is directly contributing to the strong interaction with the initiating *ATP.* Strikingly, both the *Mm*∆ZnFD deletion mutants displayed a drastically reduced primase activity, but comparable to the wild-type *Hs*PrimPol (Figure 7c, compare lanes 5, 18, and 21), thereby emphasizing the enormous difference in primase activity of these two wild-type (mouse and human) PrimPols.

### 2.7. The Zn-Finger Domain of the Mouse PrimPol Likely Interacts with the β- and γ-Phosphate Moiety of the 5′-Initiating Ribonucleotide

**Figure 8:**
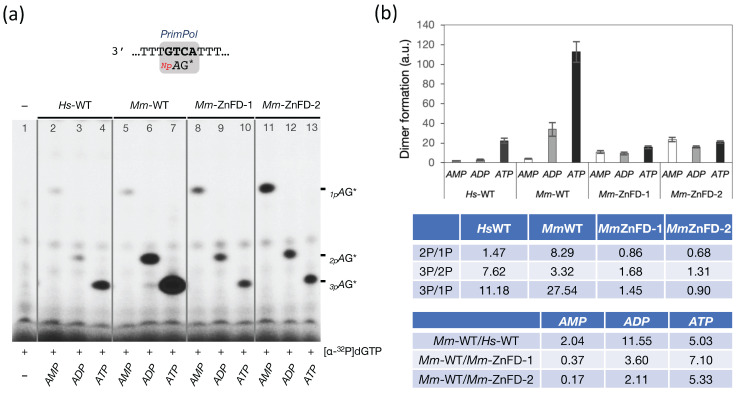
*Mm*PrimPol displays a strong preference for a 5′-nucleotide with a γ-phosphate, which requires the Zn-finger domain. (**a**) Primase assay using the 3′ T_10_-GTCA-T_15_ 5′ template to measure dimer formation by the human and mouse PrimPol (400 nM) in the presence of [α-^32^P]dGTP (16 nM) and either *AMP*, *ADP*, or *ATP* (10 µM) as the initiating nucleotide. The electrophoretic mobility of each dimer is influenced by the number of phosphates (*_1p_A*G*, *_2p_A*G*, and *_3p_A*G*) as revealed after autoradiography. (**b**) Quantification of three independent experiments as the one shown in (**a**) representing the average of the different dimers formed, according to the 5′-ribonucleotide used. Note: a.u. stands for arbitrary units derived from densitometric analysis of the autoradiographs. The dimer formation ratios among selective pair of dimers based on the number of the 5′-terminal phosphates (1P, 2P, 3P) are indicated in the table below.

As reported in Martínez-Jiménez et al., 2018 [16], the ZnFD of *Hs*PrimPol contributes to primer synthesis through the interaction with the γ-phosphate moiety of the 5′-nucleotide, which is critical during dimer formation as well as during primer maturation. To determine whether the murine ZnFD confers enhanced primase activity via a stronger interaction with the γ-phosphate moiety, we assessed dimer formation using mono-, di-, and triphosphate versions of the initiating 5′-nucleotide (*ATP*, *ADP*, or *AMP*). These assays were performed in the presence of a 3′G**TC**A template and [α-^32^P]dGTP as the 3′-site nucleotide. As depicted in Figure 8a,b, the formation of the *_3p_A*G dimers by both the human and the mouse PrimPols was maximal with *ATP* (lanes 4 and 7), consistent with the importance of the γ-phosphate moiety described by Martínez-Jiménez et al. (2018) [16]. Interestingly, while *Hs*PrimPol was just slightly affected by the presence of β-phosphate in the initiating 5′-nucleotide (compare lanes 2 and 3), dimer formation by *Mm*PrimPol was enhanced more than 8-fold when using *ADP* instead of *AMP* (compare lanes 5 and 6), being more efficient for making the *_2p_A*G* dimers (lane 6) than *Hs*PrimPol for making the *_3p_A*G dimers (lane 4). This result highlights that *Mm*PrimPol exhibits a very strong selectivity for the β- and γ-phosphate moieties of the incoming 5′-nt, as the dimer formation was enhanced 27.5-fold when using *ATP* instead of *AMP*. By contrast, *Hs*PrimPol showed only an 11.2-fold advantage with the presence of these two additional phosphates (Figure 8b).

Also consistent with Martínez-Jiménez et al. (2018) [16], our data demonstrate that the interaction of *Mm*PrimPol with the β- and γ-phosphates of the initiating nucleotide is dependent on the ZnFD. As shown in Figure 8a (lanes 8 to 13), both the *Mm*∆ZnFD deletion mutants gain no advantage of the presence of a β- or γ-phosphate moiety during dimer synthesis. Beyond losing the benefit of using an *ATP* for primer initiation, the absence of the Zn-finger domain in *Mm*PrimPol actually facilitated the use of *AMP* during primer synthesis. As shown in Figure 8a,b, both the *Mm*ZnFD mutants exhibited a 2.5- to 6-fold increase capacity to use *AMP* as the 5′-nt compared to the WT *Mm*PrimPol (compare lane 5 to lanes 8 and 11). This could indicate that the three phosphates of *ATP* could be relevant ligands for *Mm*PrimPol, but the lack of the ZnFD improves the stabilization of the α-phosphate moiety of *AMP*.

Collectively, our enzymatic characterization of *Mm*PrimPol suggests that this enzyme is optimized for primer synthesis to a greater extent than its human ortholog. This optimization is attributable to the *Mm*∆ZnFD, likely providing a stronger interaction with the initiating 5′-ribonucleoside triphosphate during primer synthesis.

### 2.8. Deletion of HsPrimPol Flexible Region (∆231–246) Alters Its DNA Polymerase-DNA Primase Balance

**Figure 9:**
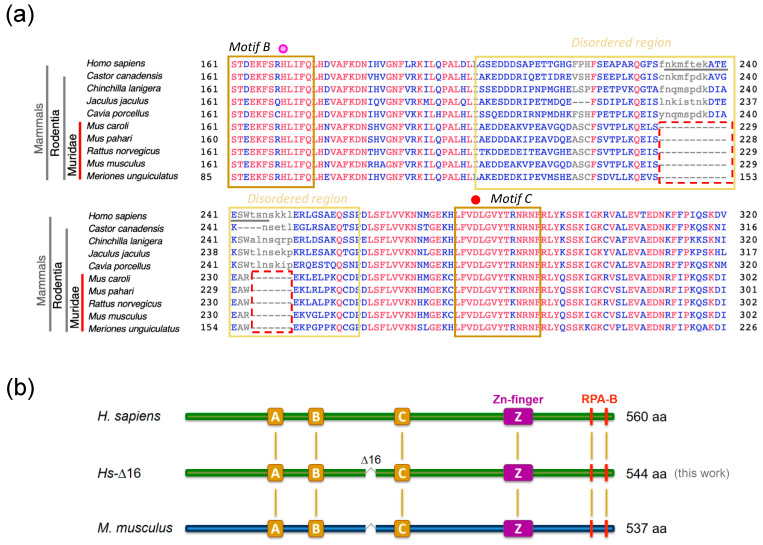
PrimPols from the Muridae family lack 18 amino acids between the catalytic motifs B and C. (**a**) Multiple amino acid sequence alignment of PrimPols from the clade of Euarchontoglires revealed that only the Muridae family has lost 11+7 amino acids (dashed red boxes) in a disordered region (light-yellow box) located between the catalytic motifs B and C. The pink dot indicates the conserved histidine that works as a 3′-nucleotide ligand residue (*motif B*) and the red dot indicates a conserved aspartate that functions as a metal ligand (*motif C*). (**b**) To mimic this deletion in *Hs*PrimPol, 16 amino acids (underlined residues 231 to 246) within the sequence of interest were eliminated, producing a protein of 544 residues (*Hs*-∆16^(∆231–246)^) that is more similar in length to *Mm*PrimPol (537 residues). The eliminated 16 amino acids in *Hs*PrimPol are located in a region spanning residues 201 to 260 (light-yellow box), that is disordered/unresolved in the crystal structure of *Hs*PrimPol [26].

The significant difference in DNA primase activity between the mouse and human PrimPol, as well as their different DNA primase/DNA polymerase balance, suggests that there is an intrinsic structural reason behind this. The multiple amino acid sequence alignment of PrimPols from diverse organisms identified a variable region situated between the catalytic motifs B and C, which exhibits moderate conservation and varies in length, being approximately 50 amino acids longer in mammals compared to insects or plants [8]. Further alignments conducted in this study (Figure 9a), more specifically of PrimPols from mammals within the Euarchontoglires clade, revealed that only the Muridae family has lost an 11 + 7 amino acid sequence within this variable region, that corresponds to amino acids 230 to 250 in *Hs*PrimPol (Figure 9a, indicated by red boxes). Interestingly, this variable region is part of a larger disordered region (residues 201 to 260) that could not be crystalized in *Hs*PrimPol [26].

**Figure 10:**
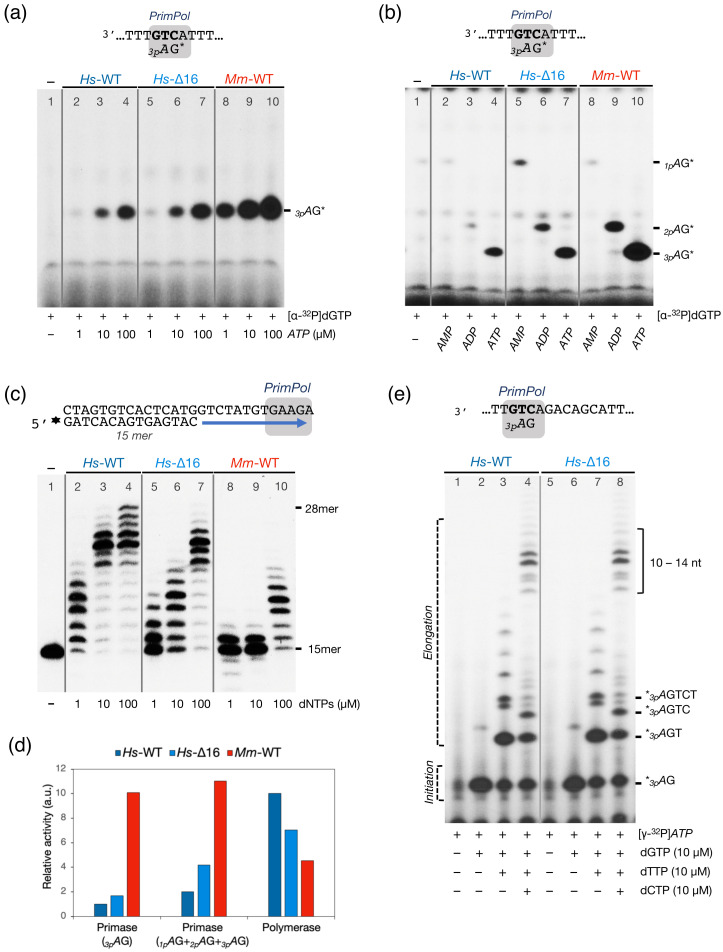
Amino acids 231 to 246 of *Hs*PrimPol are not essential for its primase and polymerase in vitro activity**.** (**a**) Dimer formation by the wild-type human and mouse PrimPols, and the deletion mutant *Hs*-∆16^(∆231–246)^ (400 nM) was assessed by providing oligonucleotide 3′ T_10_-GTCA-T_15_ 5′ (1 µM), 1 mM MnCl_2_, low concentration (16 nM) of the 3′-elongating nucleotide [α-^32^P]dGTP, and *ATP* (1, 10, and 100 µM) as the 5′-initiating nucleotide. (**b**) Competence of *Hs*-∆16 to select a 5′-nucleotide triphosphate was assessed during primase synthesis using the oligo GTCA as template, *AMP*, *ADP*, or *ATP* as the initiating 5′-nt (10 µM), [α-^32^P]dGTP at low concentration (16 nM), 1 mM MnCl_2_, and 400 nM of the indicated PrimPols. (**c**) DNA polymerase activity of the wild-type and ∆16 PrimPol were evaluated using a synthetic [γ-^32^P]-labeled primer hybridized with a template containing a 13-nt 3′-overhang, 1 mM MnCl_2_, and increasing doses of dNTPs as indicated. (**d**) Relative quantification of previous assays: primase (*_3p_A*G), quantification of dimers *_3p_A*G generated with 1 µM *ATP* in part a; primase (*_3p_A*G+*_2p_A*G+*_1p_A*G), sum of the three dimers formed either with *ATP*, *ADP*, or *AMP* in part b; polymerase, relative primer extension in the presence of 1 µM dNTPs as shown in part c. Note: a.u. stands for arbitrary units, derived from densitometric analysis of the autoradiographs. (**e**) Primer synthesis (initiation and elongation) by *Hs*PrimPol (*Hs*-WT) and *Hs*-∆16 mutant (400 nM) was assessed in the presence of unlabeled 3′ T_20_**GTC**AGACAGCAT_29_ 5′ as a template, in the presence of (16 nM) [γ-^32^P]*ATP* and unlabeled nucleotides (10 µM dGTP, dTTP, dCTP) as indicated. Both *Hs*PrimPols showed the same pattern of primer synthesis.

This variable region, and especially these 18 amino acids that were lost in Muridae, could be related to the differences in the DNA primase/DNA polymerase balance displayed by the murine and human PrimPol. For this purpose, a deletion mutant of *Hs*PrimPol lacking 16 amino acids from the region of interest (*Hs*∆16^(∆231–246)^) was generated, thereby rendering its length more similar to that of *Mm*PrimPol (Figure 9b). We first evaluated the initiation of primer synthesis (dimer formation) of the *Hs*∆16 mutant by providing the GTCA oligonucleotide as a template, [α-^32^P]dGTP as the 3′-site nucleotide, and increasing concentrations of *ATP* as the preferred 5′-site initiating nucleotide. Interestingly, formation of the initiating dimers by *Hs*∆16 was slightly higher in comparison to *Hs*PrimPol, but much lower than *Mm*PrimPol (Figure 10a). Additionally, we explored the preference of *Hs*∆16 for the number of phosphates present in the initiating 5′-nucleotide. *Hs*∆16 was slightly better than *Hs*PrimPol with any of the alternative nucleotides provided (*AMP*, *ADP*, or *ATP*), but the preference for nucleoside triphosphate (*ATP*) remained unaltered in the *Hs*∆16 mutant, and comparable in extent to *Hs*PrimPol (Figure 10b, compare lanes 2–4 vs. 5–7). These results indicated that the elimination of the 16 amino acids sequence in *Hs*PrimPol slightly improved its DNA primase activity, but did not reach the efficiency of *Mm*PrimPol (Figure 10a,b, lanes 8–10, and Figure 10d).

Next, we evaluated the DNA polymerase activity of the *Hs*∆16 mutant by providing a pre-synthesized primer hybridized to a template with increasing concentrations of the four dNTPs. Interestingly, the primer extension activity of *Hs*∆16 was reduced when compared to *Hs*PrimPol (Figure 10c, compare lanes 2–4 vs. 5–7); nevertheless, it was significantly more active than *Mm*PrimPol (Figure 10c, lanes 8–10, and Figure 10d). We further asked if the reduced polymerization of the *Hs*∆16 mutant would affect the elongation step during primer synthesis. By using the 3′ GTC-step 5′ as a template, [γ-^32^P]*ATP* as the 5′-initiating nucleotide, and dGTP, dTTP, and dCTP as the 3′-site elongating nucleotides, the mutant *Hs*∆16 was able to initiate and extend de novo primers as well as *Hs*PrimPol (Figure 10e). In conclusion, deletion of the 16 amino acids (residues 231 to 246) within the disordered region (residues 201 to 260) of *Hs*PrimPol causes a moderate alteration in the DNA primase/DNA polymerase balance, that points to a slight “murinization” of this deletion variant.

## 3. Discussion

This work presents the enzymatic characterization of *Mm*PrimPol, which revealed a much higher DNA primase activity compared to the previously described *Hs*PrimPol [8]. We show here that the robust capacity of *Mm*PrimPol to initiate DNA primer synthesis relies, for the most part, on a strong interaction with the β- and γ-phosphate moiety of the 5′-initiating ribonucleotide, which requires the ZnFD. Based on this study with *Mm*PrimPol, we can predict that the strength of interaction with the 5′-nucleotide is the main factor that will define differences in the priming efficiency of PrimPols, a prediction also extrapolatable to more conventional primases. On the other hand, *Mm*PrimPol and *Hs*PrimPol displayed similar efficiencies in other enzymatic parameters, such as ssDNA binding, template sequence recognition, and binding of 3′-elongating nucleotides in the context of priming. Paradoxically, the *Mm*PrimPol performance as a conventional DNA polymerase (extension of a pre-existing DNA primer) was very poor, when compared to *Hs*PrimPol. That inverted DNA primase/DNA polymerase balance in *Mm*PrimPol relative to *Hs*PrimPol is explained by a special difficulty of *Mm*PrimPol in accommodating a template/primer at the “initiation site”, likely due to physical interference with a constitutively closed ZnFD, which promotes a primase-ready configuration. Hence, *Mm*PrimPol is fully devoted to DNA priming.

As previously shown, while the integrity of the Zn-finger is dispensable for the DNA polymerase activity of *Hs*PrimPol, the Zn finger was essential for priming [9,15,16]; importantly, not only deletion of the entire ZnFD affected primer synthesis by PrimPol, but point mutations targeting Zn-ligands completely disrupted the primase activity of *Hs*PrimPol, and both type of mutants failed to rescue stalled forks in human cells [9]. The relevance of the ZnFD for priming is reinforced here, by demonstrating that its elimination in *Mm*PrimPol abolishes its robust DNA primase activity, but allows a better performance in conventional DNA polymerase assays. Therefore, these results also confirm that the Zn-finger domain modulates/inhibits the PrimPol DNA polymerase activity.

However, our findings contradict a previous report claiming that the Zn-finger is not essential for priming by *Mm*PrimPol [32]. It is worth mentioning that these authors used a fluorescein-labeled nucleotide (FAM-*ATP*) for the initiating 5′ site. We hypothesize that the bulky nature of FAM-*ATP* could favor its binding at the defective 5′site in the *Mm*ΔZnFD. If that artificial stabilization enables some catalysis, it explains the misleading conclusion of a dispensable ZnFD in *Mm*PrimPol. Surprisingly, Li et al. (2022) [32] did not include a comparison with the wild-type *Mm*PrimPol, that would be very useful to know if (1) the primase activity observed with FAM-*ATP* in the absence of the ZnFD was residual in comparison with the robust primase activity shown here for the wild-type *Mm*PrimPol, or (2) the wild-type *Mm*PrimPol has problems accommodating the fluorescent FAM-*ATP* at the 5′ site due to steric hindrance with the ZnFD, impeding its closed conformation with the AEP (primase configuration). Considering these points, and supported by our previous and current experimental data, we strongly conclude that the ZnDF of *Mm*PrimPol (and that of other PrimPols) is essential for its primase activity.

Another factor that may contribute to the robust primase activity of *Mm*PrimPol could reside in the variable and disordered region located between catalytic motifs B and C (amino acids 201–242). In *Hs*PrimPol, the disordered region corresponds to amino acids 201 to 260, and includes 18 extra amino acid residues that are absent in *Mm*PrimPol. It is likely that this disordered region [26] may function as a ‘flexible’ linker that connects the catalytic core with the ZnFD, allowing different configurations to modulate primase and polymerase function. As shown here, elimination of the 16 amino acids of this hypothetical linker in *Hs*PrimPol (mimicking *Mm*PrimPol), moderately affected the DNA primase/DNA polymerase balance, slightly improving the primase but inhibiting the conventional DNA polymerase activity of *Hs*PrimPol.

### 3.1. Initial Closing and Progressive Mobilization of the ZnFD During DNA Primer Synthesis: A Model

So far, our knowledge about PrimPol’s ZnFD function relies basically on in vitro and in vivo analysis of the ZnFD deficient mutants [9,15,16], but structural information about this domain remains unknown, as the current crystal structure of *Hs*PrimPol lacks the ZnFD [26]. Previous structural analysis of the human primosome revealed that the C-terminal domain of the non-catalytic p58 subunit participates in the binding and stabilization of the template and the initiating *NTP* [33,34]. Here we showed that the C-terminal ZnFD of the monomeric *Mm*PrimPol is also involved in the interaction with the initiating *NTP*, thus mimicking the function of p58; on the other hand, the catalytic core of PrimPol (as the catalytic subunit p48 of the dimeric primase) would be in charge of inserting 3′-nucleotides (dNTPs in the case of PrimPol) during both the initiation and elongation stages of primer synthesis, as it was also predicted for *Hs*PrimPol [16,35].

**Figure 11:**
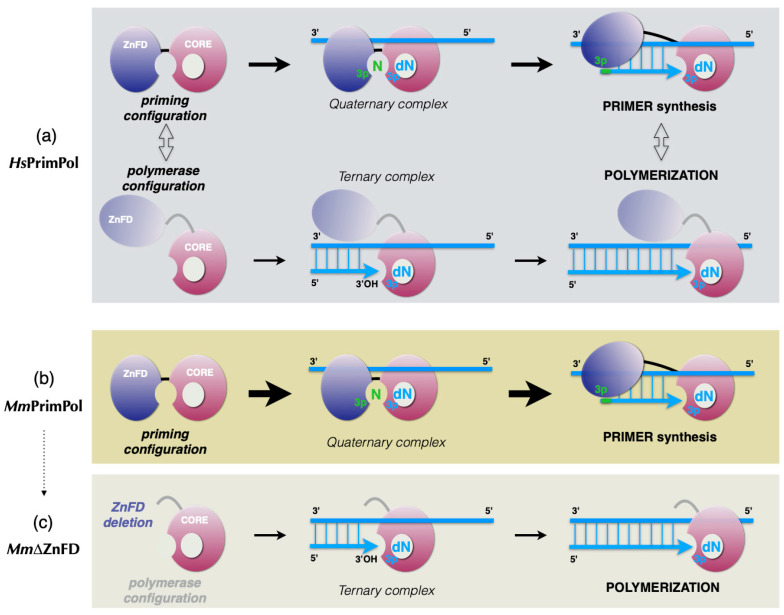
Different configurations of the ZnFD allow PrimPols to behave as a DNA primase or as a DNA polymerase. (**a**) *Hs*PrimPol can adopt both a *priming configuration* and a *polymerase configuration*, as a function of the mobilization of the ZnFD, and consequently can display both de novo primer synthesis and conventional polymerization. (**b**) The robust DNA primase activity of *Mm*PrimPol, and its very limited polymerization capacity, is explained by a unique configuration of the ZnFD, in a prevalent *priming configuration*. (**c**) Deletion of the ZnFD of *Mm*PrimPol impedes priming, but promotes an artificial *polymerase configuration* that allows conventional DNA polymerization on a template primer. See text for details.

The enzymatic characterization of *Hs*PrimPol as both a DNA primase and a DNA polymerase supports a model in which PrimPol can assume two different configurations, based on the mobilization of the ZnFD, which favor either de novo primer synthesis or conventional polymerization (Figure 11a); the priming configuration is a tight (or closed) configuration that brings close together the catalytic core, responsible for binding the ssDNA template and 3′dNTP, and the ZnFD, which is crucial for binding the 5′NTP. Thus the two domains collaborate to complete the initiating quaternary complex, followed by dimer formation and its further extension to build a mature primer; alternatively, the polymerase configuration is a loose (or open) form of PrimPol, where the catalytic core operates independently of the ZnFD, that has to be mobilized to allow the accommodation of a primer–template substrate close to the catalytic site and the incoming 3′dNTP (ternary complex) to sustain normal (DNA polymerase-like) polymerization of dNTPs onto the primer strand.

The enzymatic characterization of *Mm*PrimPol reported here supports that the murine PrimPol has a more prevalent priming configuration, that explains its robust DNA primase activity, in detriment to polymerization (Figure 11b). Thus, *Mm*PrimPol seems to be pre-configured to start primer synthesis by assuming a constitutive and tightly closed *priming configuration* likely due to a more defined and closed positioning of its ZnFD. Importantly, the elongation phase of primer synthesis, that requires progressive mobilization of the ZnFD is not compromised in *Mm*PrimPol. In agreement with these conclusions, an effective DNA polymerase activity was unmasked in *Mm*PrimPol when the ZnFD was eliminated, leaving the protein in an artificially opened configuration that licenses polymerization (Figure 11c).

Future crystal structures of the entire PrimPol in priming configuration will be of great value to describe how the ZnFD contributes to stabilize the initiating 5′-nucleotide, which specific residues are involved in the interaction with its sugar and phosphate residues, and how these interactions are modified or remain during processive elongation to generate a mature primer. The robust DNA primase activity of *Mm*PrimPol, and in particular its constitutive priming configuration, with a less mobile ZnFD, make *Mm*PrimPol an ideal candidate for crystallization in primase mode.

### 3.2. Mouse PrimPol: Need for a Strong Primase?

The significant differences in the specific activity of the human and mouse PrimPols raise the question of why mice have evolved such a potent DNA primase. A brief look into these two species gives a clue to explain the need for a robust priming by *Mm*PrimPol. Some physiological characteristics of humans and mice are very contrasting, such as body weight and size, thermoregulation, lifespan, reproductive rate, aging processes, and rapid cellular turnover, just to list a few. Many of these features have close connections to metabolism, which directly correlates with mitochondrial function [36]. Thus, preserving the integrity of the mitochondria is very important to keep the homeostasis in the physiology of the entire organism. In fact, the metabolic rates between these two species vary enormously, with the level of oxidative stress and damage being higher in mice.

It is known that the lack of PrimPol in mice results in a slight shortening of lifespan, increased incidence of late-onset tumor development, higher sensibility to UV-light injury, and impaired mitochondrial function (unpublished data). In this context, PrimPol has been shown to contribute to the maintenance of both nuclear and mitochondrial genomes through its repriming activity, that allows the completion of DNA replication under many different stress conditions [9,24,25,28]. Thus, it is conceivable that selection of a murine PrimPol with a robust primase activity could be an adaptive response to its high metabolic rate, elevated ROS production, and rapid cellular turnover. So, we speculate that the potent primase activity of *Mm*PrimPol would meet the need to restart stalled forks in a more demanding cellular environment.

## 4. Materials and Methods

### 4.1. Reagents

Inorganic salts, acids, bases, and organic compounds were purchased from Merck (Darmstadt, Germany), Sigma (St. Louis, MO, USA), and AppliChem (Darmstadt, Germany). The chromatography resins for protein purification used in this work were Ni-NTA Superflow purchased from Qiagen (Hilden, Germany) and Heparin Sepharose Fast Flow purchased from GE Healthcare (Fairfield, CT, USA).

Unlabeled ultrapure *AMP*, *ADP*, *NTPs*, and dNTPs were supplied by GE Healthcare (Fairfield, CT, USA). Labeled nucleotides, [γ-^32^P]*ATP* and [α-^32^P]dGTP, were purchased from Revvity (Waltham, MA, USA). T4 polynucleotide kinase, T4 DNA Ligase, and DNA restriction endonucleases were obtained from New England Biolabs (Ipswich, MA, USA) and Roche (Basel, Switzerland). Vent polymerase was supplied by New England Biolabs (Ipswich, MA, USA).

### 4.2. Oligonucleotides

DNA oligonucleotides were synthesized by Sigma Aldrich (St. Louis, MO, USA) and purified by 8 M urea-20% polyacrylamide gel electrophoresis.

### 4.3. Cloning the Mouse PrimPol and Mutant Generation

*Mm*PrimPol is codified by the *CCDC111* gene, with the same name as its human ortholog, and it is located in chromosome 8 (8 B1.1). A full-length cDNA of the *CCDC111* mouse gene, cloned in the pET28/*murineCCDC111* vector was kindly given by Dr. Raimundo Freire (Hospital Universitario La Laguna, Canary Islands, Spain). The *CCDC111* gene was amplified by PCR using oligos *Mm*WT-forward (5′-ACGATCATATGTTGAGGAAATGGGAGGCAAG-3′) and *Mm*WT-reverse (5′-ACGATGGATCCTCAGCTATTCTGTAAAGCTTCTATA-3′), that introduce the *Nde*I-*BamH*I sites, respectively, then PCR products were digested and introduced in pET16b expression vector (Novagen, Darmstadt, Germany) cut with the same restriction enzymes, as already described for *Hs*PrimPol [8]. The resulting plasmid was named pET16/*murineCCDC111* and kept in an *E. coli* DH5α strain. Two deletion mutants of *Mm*PrimPol lacking the C-terminal domain including the ZFD were generated, named *Mm-*∆ZnFD-1^(1–391)^ and *Mm-*∆ZnFD-2^(1–336)^. The deletion mutants were constructed by PCR amplification of the first 1173 nucleotides (*Mm-*∆ZnFD-1) or 1008-nt (*Mm-*∆ZnFD-2) of the mouse *CCDC111* cDNA using primers *Mm-*WT-forward combined with *Mm-*∆ZnFD-1-reverse (5′-ACGATGGATCCTCACAGTAATTCTTCCGGGAAAAAG-3′) or *Mm-*∆ZnFD-2-reverse (5′-ACGATGGATCCTCACTTTCGTTTAGTCTGAGATGGG-3′). The amplified products were inserted in *E. coli* expression plasmid pET16 (Novagen) in *Nde*I-*Bam*HI sites and further sequenced to confirm the deletion version of *Mm*PrimPol.

### 4.4. Purification of the Human and Mouse PrimPol Variants

Protein expression and purification was performed identically for the human and mouse PrimPol, both the wild-type and the mutant variants. The protocol for purification was as described previously [8].

### 4.5. Polymerase Assay on Specific Primer: Template Molecules

DNA polymerase assays were carried out with labeling at the 5′ the primer using PNK and [γ-^32^P]*ATP* (250 µCi, 3000 Ci/mmol), then hybridized to a complementary template in a buffer containing 0.3 M NaCl, 50 mM Tris-HCl pH 7.5. DNA sequences are indicated in the figures. Reaction mixtures (in 20 µL) contained Buffer R [50 mM Tris–HCl pH 7.5, 40 mM NaCl, 2.5% (*w*/*v*) glycerol, 1 mM DTT, 0.1 mg/mL BSA, 1 mM MnCl_2_, or 5 mM MgCl_2_], 2.5 nM [γ-^32^P]-labeled primer–template, 200 nM purified PrimPol variant, and dNTPs or *NTPs* as indicated in each figure. Polymerase assays were incubated 20 min at 30 °C, and stopped by adding 8 μL of formamide loading buffer (95% formamide, 20 mM EDTA, 0.1% xylene-cyanol, and 0.1% bromophenol blue), then loaded onto an 8 M urea-containing 20% polyacrylamide sequencing gel. Primer extension was detected by autoradiography and the images analyzed with ImageJ software 1.49v.

### 4.6. Primase Assay on Specific Oligonucleotide Templates

Primase assays were carried out using the indicated ssDNA oligonucleotide, containing the priming site 3′**GTC 5′** flanked by thymidine tracts or variations around this sequence. The reaction mixture (20 µL) contained Buffer R, 1 µM ssDNA template, 400 nM PrimPol, 16 nM [γ-^32^P]*ATP* or [α-^32^P]dGTP (250 µCi, 3000 Ci/mmol) and the nucleotides indicated in each figure at the described concentrations. After incubation for 30 min at 30 °C, reactions were stopped by adding 8 μL of formamide loading buffer (95% formamide, 20 mM EDTA, 0.1% xylene-cyanol, and 0.1% bromophenol blue) and the products resolved in an 8 M urea-containing 20% polyacrylamide sequencing gel (50 cm long) that was run at 50 W for 2 h. Following electrophoresis, de novo synthesized primers were detected by autoradiography and the images were analyzed with ImageJ software 1.49v.

### 4.7. EMSA for PrimPol/ssDNA Binary Complex

The PrimPol/ssDNA binding assay was performed with [γ-^32^P]-labeled ssDNA oligonucleotide (3′T_20_-GTCAGACAGCA-T_29_ 5′). The reaction mixtures (20 µL) contained Buffer R, 2.5% (*w*/*v*) PEG-4000 and purified PrimPol in the concentrations indicated in the figure. Samples were pre-incubated for 10 min at 4 °C and then incubated for 10 min at 30 °C with 2.5 nM 5′-labeled ssDNA. Reactions were stopped by adding 2 µL loading Buffer S (50% glycerol, 0.1% (*w*/*v*) xylene cyanol, and 0.1% (*w*/*v*) bromophenol blue), and analyzed in a native 6% polyacrylamide gel, resolved in Tris-glycine buffer (pH 8.3) at 180 V for 120 min at 4 °C. After electrophoresis, gels were vacuum-dried and mobility shift of free ssDNA versus enzyme/ssDNA complex was analyzed by autoradiography.

### 4.8. EMSA for PrimPol/ssDNA/dNTP Pre-Ternary Complex

Pre-ternary (PrimPol/ssDNA/dG) complex formation using PrimPol variants and [α-^32^P]dGTP (16 nM) were evaluated in Buffer D (50 mM Tris–HCl pH 7.5, 40 mM NaCl, 2.5% (*w*/*v*) glycerol, 1 mM DTT, 0.1 mg/mL BSA), supplemented with 0.5 µM ssDNA 3′ T_20_-**GTCC**-T_36_ 5′, and 1 mM MnCl_2_. Reactions (20 µL final volume) were incubated for 10 min at 30 °C. Then, 2 µL of loading Buffer S was added and the reactions were analyzed as described above.

## Data Availability

Not available.

## Abbreviations

The following abbreviations are used in this manuscript:

*Hs*PrimPol SOFTWARE VERSION INCLUDED	*Homo sapiens* PrimPol
*Mm*PrimPol	*Mus musculus* PrimPol
AEP	Archaeo-Eukaryotic Primases
ZnFD	Zinc Finger Domain
dNTPs	deoxynucleoside triphosphates
*NTP*s	ribonucleoside triphosphates
